# Brain Organoids to Evaluate Cellular Therapies

**DOI:** 10.3390/ani12223150

**Published:** 2022-11-15

**Authors:** Ana Belén García-Delgado, Rafael Campos-Cuerva, Cristina Rosell-Valle, María Martin-López, Carlos Casado, Daniela Ferrari, Javier Márquez-Rivas, Rosario Sánchez-Pernaute, Beatriz Fernández-Muñoz

**Affiliations:** 1 Unidad de Producción y Reprogramación Celular (UPRC), Red Andaluza de Diseño y Traslación de Terapias Avanzadas (RAdytTA), Fundación Progreso y Salud, 41092 Sevilla, Spain; 2 Departamento de Farmacia y Tecnología Farmacéutica, Facultad de Farmacia, Universidad de Sevilla, 41012 Sevilla, Spain; 3 IBiS, Instituto de Biomedicina de Sevilla, 41013 Sevilla, Spain; 4 Department of Biotechnology and Biosciences, University Milan-Bicocca, 20126 Milan, Italy; 5 Departamento de Neurocirugía, Hospital Virgen del Rocío, 41013 Sevilla, Spain

**Keywords:** brain organoids, cell therapy, neural stem cells, neural progenitors, translation, 3 Rs, reduction

## Abstract

**Simple Summary:**

Animal models are routinely used in pre-clinical studies to evaluate the safety and efficacy of novel therapies, such as cell transplantation, but have limited predictive value. In this study, we set up an experimental model using human stem cells grown in 3D, which form rudimentary brain structures in vitro, called brain organoids. We investigated the possibility of using these brain organoids to evaluate the safety of a cell therapy product, by comparing the results obtained in our model with the standard mouse model. Our results suggest that brain organoids can be informative in the evaluation of cell therapies, helping to reduce the number of animals used in regulatory studies.

**Abstract:**

Animal models currently used to test the efficacy and safety of cell therapies, mainly murine models, have limitations as molecular, cellular, and physiological mechanisms are often inherently different between species, especially in the brain. Therefore, for clinical translation of cell-based medicinal products, the development of alternative models based on human neural cells may be crucial. We have developed an in vitro model of transplantation into human brain organoids to study the potential of neural stem cells as cell therapeutics and compared these data with standard xenograft studies in the brain of immunodeficient NOD.Cg-*Prkdc^scid^ Il2rg^tm1Wjl^*/SzJ (NSG) mice. Neural stem cells showed similar differentiation and proliferation potentials in both human brain organoids and mouse brains. Our results suggest that brain organoids can be informative in the evaluation of cell therapies, helping to reduce the number of animals used for regulatory studies.

## 1. Introduction

Cell therapies are perceived as medicines of the future and many pharmaceutical companies are now investing in the development of advanced therapies to treat different pathologies that to date have no cure [1]. However, for their translation into clinical practice, these therapies face numerous challenges such as their high cost, regulatory requirements for authorization and lack of predictive in vivo models to achieve effective translation [2,3].

For the development of cell therapeutics, a regulatory requirement is to perform efficacy and safety studies. These experiments are usually performed in animal models, most often in murine models, and may include homologous and heterologous models. Homologous studies in which mouse cells are transplanted in mouse models of the disease generally use mice with a C57BL/6J genetic background [4,5]. Heterologous models are required to test the proliferation and differentiation potential of the cell therapeutic that will be used in humans and are commonly evaluated in nude and NSG immunodeficient mice to avoid rejection of human cells [6,7,8]. These animal models have many limitations and it has been shown that results from mice experiments are rarely predictive of the human outcome [9]. These problems are even more accentuated in translational neuroscience research, since the brain structure lacks much complexity in rodents and fails to mimic many key features of the human brain [10,11]. Non-human primates can provide more relevant information because of their homology to humans. However, high costs and the need for special animal facilities, among other limitations, hinder generalization of experiments with these animals. In addition to the lack of predictability, animal experiments are expensive and laborious, and there is an increasing demand from society and regulatory bodies to reduce the number of animals used for research [11].

In vitro models based on human cells cultured in 2D have been proposed as alternative systems to test specific safety and efficacy attributes of new therapeutics. However, 2D cultures also have important limitations. Growing cells as monolayers can lead to alterations in the cytoskeleton, nucleus and cell shape, resulting in altered gene expression patterns [12,13]. Furthermore, 2D cultures fail to reproduce the 3D nature of in vivo environments and lack crucial cell–cell as well cell–matrix interactions, limiting their usefulness [14,15]. Therefore, for an effective translation of new cell therapies to the clinic, the development of more complex and predictive human models is crucial.

In recent years, alternative 3D in vitro models, which could replace the animal model in certain studies have been developed. Among these, the most promising are organoids, which are stem cell-derived mini-organs developed in the laboratory [16]. The emergence of organoids has generated tremendous interest in the field of regenerative medicine [17]. Brain organoids are 3D in vitro aggregates generated from human induced pluripotent stem cells (hiPSC), or human embryonic stem cells (hESC) that reproduce specific brain structures simulating different human brain regions in their composition and cytoarchitecture [18]. They produce neural stem cells (NSC) that develop into mature cerebral cell types and recapitulate both the transcriptome and the epigenome of the fetal brain [19,20]. In this way, brain organoids allow obtaining in vitro information about the human brain to study brain development and cellular interconnections, and are emerging as an excellent in vitro model for the investigation of mechanisms involved in neurological diseases and for drug testing [21]. For example, cortical organoids have been successfully used as drug screening tools to test different chemical drugs for Rett Syndrome [22,23]. However, the applicability of organoids for the evaluation of stem cell-based therapeutics has not been explored to date.

NSC are multipotent cells that can potentially differentiate into neurons and glial cells and secrete trophic and immunomodulatory factors. All these properties make NSC attractive for regenerative therapies and several clinical-grade NSC-based medicinal products have been tested in clinical trials [24]. Evidence of their safety and efficacy profiles has been collected in all those studies from experiments performed in immunodeficient animals, mostly rodents.

Here, we have investigated whether human brain organoids can be used as in vitro models for the evaluation of human cell-based therapeutics. We successfully transplanted human NSC into brain organoids and compared these data with standard xenograft studies in the brain of NOD.Cg-*Prkdc^scid^ Il2rg^tm1Wjl^*/SzJ (NSG) mice. Our results show that brain organoids can be informative when exploring human NSC therapeutic potential, helping to facilitate preclinical studies and to reduce the number of animals used for regulatory studies.

## 2. Materials and Methods

### 2.1. Organoid Generation

The hiPSC line CBiPSS1sv-4F-5, derived from CD133^+^ umbilical cord cells (CB-hiPSC), was used to generate cortical organoids. This cell line is available from the Spanish National Repository, and data regarding characterization can be downloaded at https://eng.isciii.es (accessed on 1 November 2022). The use of CB-hiPSC was approved by the Andalusian Ethical Committee of Research with Biological Samples of an Embryonic Origin and Similar Cells (PR-02-2016). CB-hiPSC were maintained on Matrigel-coated plates, cultured in mTeSR^TM^ Plus medium (Stemcell Technologies, Vancouver, British Columbia, Canada, Cat. #100-0276). Normal karyotype and pluripotency were verified before organoid generation (Figure S1A,B).

**Figure 1:**
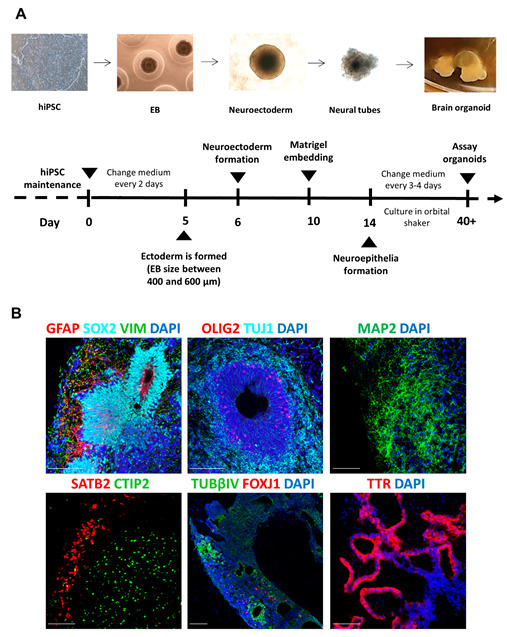
**Generation of human brain organoids.** (**A**) Protocol used for the generation of brain organoids. EB: embryoid body; hiPSC: human induced pluripotent stem cells. (**B**) Formation of brain structures was assessed by detection of different markers by immunofluorescence: Glial fibrillary acidic protein (GFAP), SOX2, Vimentin (VIM), OLIG2, Beta-III-tubulin (TUJ1), MAP2, SATB2, CTIP2, Tubulin-beta-IV (TUBβIV) and transthyretin (TTR). Scale bar: 100 µm.

Brain organoids were generated and cultured following a protocol slightly modified from that described by Lancaster et al. [19,25] (Figure 1A). Briefly, on day 0, 3000 hiPSC/well were seeded in mTeSR^TM^ plus medium with 10 µM ROCK inhibitor Y-27632 (Tocris Bioscience, Minneapolis, MN, USA, Cat. 1254) in agarose microwells to generate uniform embryoid bodies (EB). Agarose microwells were prepared with a Micro Tissues 3D Petri Dish spheroids micro-mold (size L, 9 × 9 array; Sigma, St. Louis, MO, USA, Cat. #Z764019-6EA) with 2% D1 Low EEO agarose (Pronadisa by Conda, Torrejón de Ardoz, Madrid, Spain, Cat 8010.22) in Dulbecco’s PBS without CaCl_2_ and MgCl_2_ (Sigma, St. Louis, MO, USA, Cat. D8537). The solidified microwells were equilibrated with 2.5 mL mTeSR^TM^ plus medium for 15 min at 37 °C before use. For 6 days, EB were fed every other day with mTeSR^TM^ plus medium without ROCK inhibitor and then changed to neural induction media (NIM) containing Dulbecco’s modified Eagle Medium (DMEM):F12 (Gibco by Thermo Fisher Scientific, Waltham, MA, USA, Cat. 21331020), 1X N2 supplement (Gibco by Thermo Fisher Scientific, Waltham, MA, USA, Cat. 17502-048), Glutamax (Gibco by Thermo Fisher Scientific, Waltham, MA, USA, Cat. 35050-061), minimum essential media–non essential amino acids (MEM-NEAA) (Sigma, St. Louis, MO, USA, Cat. P4999) and 1 µg/mL heparin 1000 UI/mL (Rovi, Madrid, Spain, Cat. 641747). Then, EB were fed every other day with NIM for 4–5 days. On day 10 of the protocol, organoids were covered with Matrigel (Corning, New York, NY, USA, Cat. 354277) and grown in differentiation media containing a 1:1 mixture of DMEM/F12 and Neurobasal (Gibco by Thermo Fisher Scientific, Waltham, MA, USA, Cat. 21103-049) with 1X N2 supplement, 2X B27 supplement without vitamin A (Gibco by Thermo Fisher Scientific, Waltham, MA, USA, Cat. 12587-10), 1X Glutamax, 1X MEM-NEAA, 1X P/S (Sigma, St. Louis, MO, USA, Cat. P4333), 0.09% 2-mercaptoethanol (Gibco by Thermo Fisher Scientific, Waltham, Massachusetts, USA, Cat. 21985-023) and 0.025% insulin (Sigma, St. Louis, MO, USA, Cat. I9278). After 4 days of stationary growth, the organoids were transferred to an orbital shaker (Celltron, Infors HT, Bottmingen, Switzerland) installed in a 37 °C incubator with differentiation media, but adding B27 supplement with vitamin A (Gibco by Thermo Fisher Scientific, Waltham, MA, USA, Cat. 17504-044).

### 2.2. Transplantation into Brain Organoids

For transplantation experiments, we used human NSC isolated from the germinal zone of the ventral forebrain (Gz-NSC) and purified for the expression of the stem cell marker CD133^+^ [26]. For transplantation, two different NSC lines were first expanded and transduced with a lentiviral vector with the green fluorescent protein containing a nuclear localization sequence (EGFP-NLS) under the constitutive promoter of spleen focus-forming virus (SFFV). NLS allows EGFP to enter in the nucleus of NSC facilitating the quantification of transplanted cells. Lentiviral particles were pseudotyped with the VSVG protein (vesicular stomatitis virus G protein). Viral particles were added to the culture medium at a concentration of 25,000 TU/mL (transduction units/mL) for 6 h. Subsequently, the medium was removed and EGFP expression was confirmed under a fluorescence microscope at 24–48 h.

For cell transplantation, brain organoids were selected and individually transferred to a low-adhesion 24-well plate without culture medium. Brain organoids were injected under a stereomicroscope (SMZ1500, Nikon, Tokyo, Japan) located inside a laminar flow cabinet using a Hamilton syringe with a 30GA small Hub RN sterile needle. Then, 1 μL of cell suspension (1 × 10^5^ NSC-EGFP in hypothermosol (HTS, Biolife Solutions, Bothell, WA, USA Cat. 101102) was slowly injected (0.25 μL/30 seg). At least 3 brain organoids were transplanted with each of the NSC-EGFP lines and with HTS alone (1 μL) to control for background fluorescence. After transplantation, medium was slowly added to the wells and organoids were maintained 24 h without agitation. Then, they were cultured again under agitation until the end of the experiment. The presence of fluorescent transplanted cells (NSC-EGFP^+^) inside the brain organoids was evaluated using a Nikon Eclipse Ti-S inverted fluorescence microscope at different time points after transplantation.

Three weeks after the transplant, brain organoids were fixed with 4% paraformaldehyde (PFA, Santa Cruz Biotechnology, Dallas, TX, USA, Cat. sc-281692) for 20 min at room temperature (RT), equilibrated in 30% sucrose (VWR, Radnor, PL, USA, Cat. M117-1KG) in phosphate buffer saline (PBS, Gibco by Thermo Fisher Scientific, Waltham, MA, USA, Cat. 14190-144), embedded in optimal cutting temperature compound (OCT, Sakura Finetek Inc., Torrance, CA, USA, Cat. 4583) and kept at −80 °C. Once frozen, serial sections of 20 µm thickness were cut in the cryostat (CM 3050 S, Leica, Wetzlar, Germany) and processed for immunofluorescence analysis. At least 2 organoids transplanted with each NSC line were analyzed.

### 2.3. Transplantation into the Brain of Immunodeficient Animals

Animals were anesthetized by inhalation with sevoflurane (5% sevoflurane, 2% oxygen) in an anesthesia induction chamber and were later transferred to a stereotaxic frame where 3 μL of cell suspension (3 × 10^5^ NSC in HTS) were injected into the caudate-putamen (anteroposterior: 0 mm; dorsolateral: −2.5 mm from bregma; dorso-ventral −3 mm from dura matter) [27] close to the lateral ventricle. In total, 8 animals were transplanted with each of the NSC lines. Three weeks after transplantation, animals were anesthetized by injection of a sublethal dose of 30–40 mg/kg of thiobarbital intraperitoneally and transcardially perfused with a saline solution followed by 4% PFA diluted in cold PBS. The brains were removed and post-fixed in 4% PFA overnight, cryoprotected in 30% sucrose in PBS and then, embedded in OCT and kept at −80 °C. Once frozen, 20 µm-thick serial sections were cut in the cryostat and immunofluorescence studies were carried out. Expression of the different markers was investigated in at least 2 animals transplanted with each NSC line. Animal care and experimental procedures were conducted according to the current National and International Animal Ethics Guidelines and approved by the Research Ethics Committee of University Hospital Virgen Macarena and Virgen del Rocío (0287-N-20).

### 2.4. Immunofluorescence

Immunofluorescence experiments were performed on mouse brains and human brain organoids serial sections, which were permeabilized and blocked in PBS with 10% Donkey Serum (DKS, Sigma, St. Louis, MO, USA, Cat. D9663) and 0.1% Triton X-100 (Sigma, St. Louis, MO, USA, Cat. T8787) for 90 min. An antigen retrieval step with 10mM citrate buffer (Thermo Fisher Scientific, Waltham, Massachusetts, USA, Cat. J63950) was performed previously to overnight incubation with the primary antibodies at 4°C. Samples were subsequently incubated with secondary antibodies and Hoechst for 1 h at RT and mounted with ProLong™ Gold Antifade Mountant (Thermo Fisher Scientific, Waltham, MA, USA, Cat. P36930). Primary and secondary antibodies are listed in Table S1. Acquisition of fluorescence images was performed in a Nikon Eclipse Ti-S or a Leica TCS-SP5 confocal microscope. Images were processed using Image J 1.53t software developed at the National Institutes of Health and the Laboratory for Optical and Computational Instrumentation (LOCI, University of Wisconsin, Madison, WI, USA) [28]. For cell counting, at least 100 transplanted cells were counted for each condition.

### 2.5. Statistics

Data are represented as mean ± standard deviation (SD). Significance was determined using two-tailed Student’s *t* test for comparisons between two groups. A two-way analysis of variance (ANOVA) was used to compare cell proliferation of each line in the two model systems (mouse brain versus human brain organoids). *p* < 0.05 was considered significant. All statistical analyses were performed using the GraphPad Prism 8.01 software (San Diego, CA, USA).

## 3. Results

### 3.1. Transplantation into Human Brain Organoids

We generated brain organoids from hiPSC following the original protocol designed by Lancaster and colleagues [19,25] with few modifications, the most important of them being the use of agarose wells to favor uniform EB formation (Figure 1A).

We maintained organoids for 1–2 months in culture. The presence of several human brain tissues and cell types was characterized by immunofluorescence analysis. We identified structures resembling the ventricular zone (VZ) with many NSC positive for the stem cell transcription factor SOX2 and relatively more mature cell types located in the outer part of the VZ, such as neuroblasts positive for doublecortin (DCX) or neuron specific beta III tubulin (TUJ1), precursors of oligodencrocytes positive for OLIG2 and more mature neurons positive for the neuronal marker, microtubule-associated protein 2 (MAP2). We also detected more mature structures such as cerebral cortex, with early-born and late-born cortical neurons identified by the transcriptional regulators SATB2 and CTIP2 forming rudimentary layers, ependyma (positive for TUBβIV and FOXJ1) and non-neuronal cuboid epithelia characteristic of the choroid plexus, marked by the expression of transthyretin (TTR) (Figure 1B). We next analyzed several markers expressed during human brain development at two time points. We found that the expression of the early forebrain marker FOXG1 and the NSC marker SOX2 decreased with time, while the expression of the neuroblast markers DCX and GAD increased with time, indicating progressive maturation of cells in the brain organoids (Figure S1C).

**Figure 2:**
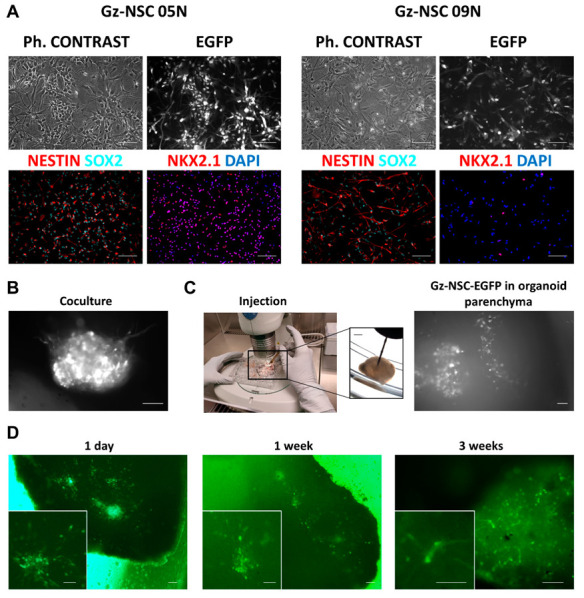
**Gz-NSC transplantation in brain organoids.** (**A**) Gz-NSC were transduced with a lentiviral vector containing EGFP with a NLS to allow the detection of transplanted cells in the human tissue. Upper panels show phase contrast images and fluorescent images of EGFP-NLS. Lower panels show expression of the NSC markers Nestin and SOX2 and the ventral regional transcription factor NKX2.1 which is expressed at the level of the medial ganglionic eminence. The two Gz-NSC lines used for transplantation experiments expressed different levels of NKX2.1. Scale bar: 100 µm. (**B**) Coculture of Gz-NSC-EGFP and brain organoids. Gz-NSC EGFP form a neurosphere that fuses with the organoid. Scale bar: 100 µm. (**C**) Cell injection under a stereomicroscope inside a laminar flow cabinet. Scale bar: 100 µm and 1 mm in the inset (**D**) Detection of injected EGFP^+^ cells at different time points under an inverted fluorescence microscope. Scale bar: 100 µm and 50 µm in the insets.

For transplantation experiments, we used human NSC derived from the germinal zones (Gz-NSC) of the developing human brain. These cells have been purified for the expression of the stem cell marker CD133^+^ and have been extensively characterized by us [26]. Gz-NSC lines isolated from different donors showed different levels of expression of the ventral marker NKX2.1, depending on the gestational age of the donor. For this study, we selected a line with high expression of NKX2.1 and another line with low expression of NKX2.1 to investigate whether results were independent of the line differentiation bias. Before transplantation, we transduced the two lines of Gz-NSC with a lentiviral vector containing EGFP with an NLS in order to detect the grafted cells in the organoid. Gz-NSC were effectively transduced, and we verified that after transduction, they did not lose the expression of the NSC markers Nestin and SOX2 and maintained their inherent regional identity determined by the expression of NKX2.1 (Figure 2A).

For cell transplantation into brain organoids, we evaluated two technical options: (1) organoid–cell coculture, where the Gz-NSC forms a neurosphere that fuses with the surface and cells slowly penetrate the organoid (Figure 2B); (2) injection into the organoid parenchyma (Figure 2C).

For this study we decided to perform the injection into the organoid parenchyma because it can help cell integration and it is more similar to the procedure in the animal model. We injected the two Gz-NSC lines transduced with EGFP in 3-month-old organoids. The brain organoids generated with our protocol were around 5 mm in size and injection was easily performed with a Hamilton microsyringe under a stereomicroscope (Figure 2C). The organoids were individually located in wells of a 24-well low binding plate without medium and injections were performed by gently immobilizing the organoid against the wall of the well (Figure 2C). Some organoids were mock transplanted with vehicle to control for background fluorescence. After injection, medium was slowly added, and organoids were maintained for 24 h without agitation to favor recovery after the puncture. In preliminary studies, we slowly injected 3 µL per organoid, but the organoids collapsed and most of the fluid came out. In this study, we injected 1 µL and there was no evident reflux, and the organoid structure was maintained with no visible signs of damage. However, 24 h after injection, we could identify some neurospheres floating around the organoid, indicating that some cells were ejected out of the organoid. Using live cell imaging, we confirmed that NSC-EGFP^+^ injected into brain organoids survived, and were integrated within the human tissue (Figure 2D). We detected cells in all transplanted organoids 24 h after cell injection, but 3 weeks later, NSC-EGFP^+^ cells were visible in only 61% (19/31) of all transplanted organoids.

**Figure 3:**
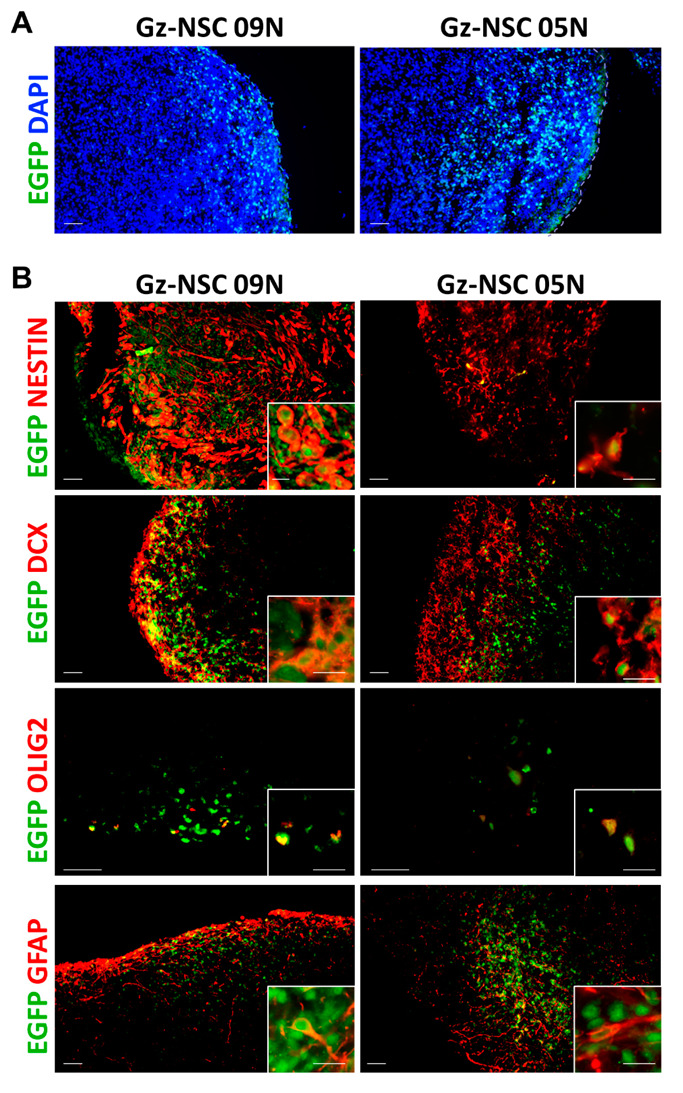
**Gz-NSC survive and differentiate in the human brain organoids.** (**A**) Survival and localization of two different lines of human Gz-NSC (EGFP^+^) transplanted into brain organoids. (**B**) Some transplanted cells maintain the stem cell phenotype as shown by the expression of Nestin while other cells differentiate into doublecortin (DCX)^+^ neuroblasts, OLIG2^+^ oligodendrocyte precursors and glial fibrillary acid (GFAP)^+^ astrocytes. Insets show colocalization of EGFP in green and the different markers in red. Scale bar: 50 µm and 20 µm in the insets.

Analysis by immunofluorescence 3 weeks after transplantation confirmed that cells from both donor cell lines survived and integrated into the organoids (Figure 3A). Injected cells mostly differentiated into DCX^+^ neuroblasts with some OLIG2^+^ oligodendrocyte precursor cells and few GFAP^+^ astrocytes, while other cells maintained an immature, Nestin^+^ NSC phenotype (Figure 3B).

### 3.2. Transplantation into Immunodeficient Mouse Brains

We compared the data from transplantation experiments into brain organoids with data obtained from standard transplantation studies of the same two Gz-NSC lines into the brains of adult NSG mice.

**Figure 4:**
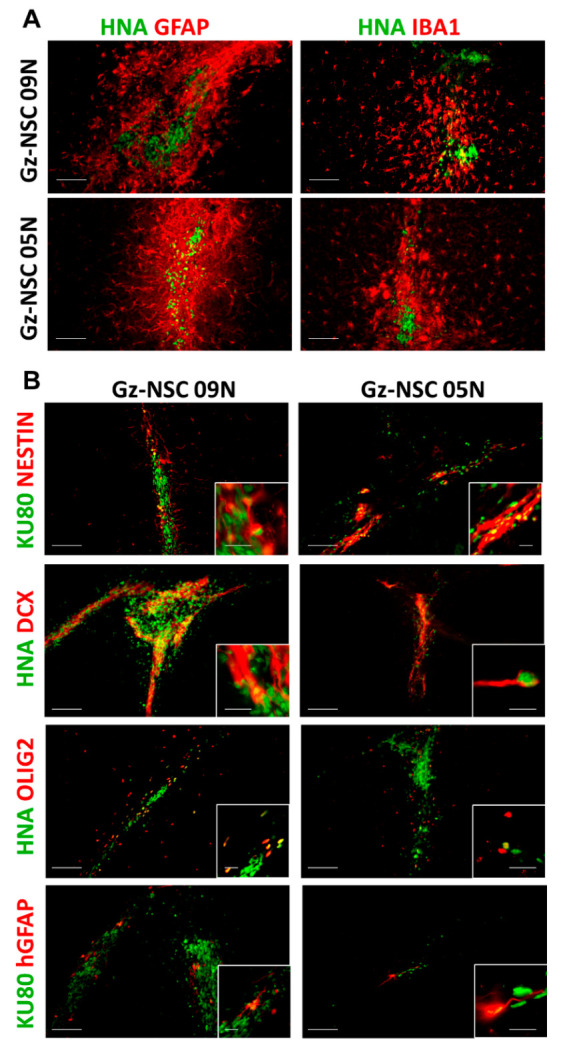
**Gz-NSC survive and differentiate in the brain of immunodeficient mice.** (**A**) Transplantation site of two different lines of human Gz-NSC identified by the expression of human nuclear antigen (HNA in green), surrounded by mouse reactive GFAP^+^ astrocytes and IBA1^+^ microglia (in red). (**B**) Some transplanted cells maintain a stem cell phenotype as shown by the expression of Nestin while other cells differentiate to DCX^+^ neuroblasts, OLIG2+ oligodendrocyte precursors and human specific (h)GFAP^+^ astrocytes. Insets show colocalization of nuclear human markers, HNA or KU80 respectively, in green, with the differentiation markers in red. Scale bar: 100 µm and 20 µm in the insets.

In mice brains, Gz-NSC mostly remained at the transplantation site, which was surrounded by reactive astrocytes positive for GFAP and activated microglia positive for IBA1 (Figure 4A). Three weeks after transplantation, Gz-NSC transplanted into the mouse brain, mostly differentiated into DCX^+^ neuroblasts with some OLIG2^+^ oligodendrocyte progenitor cells and few GFAP^+^ astrocytes, with some cells still maintaining a more immature Nestin^+^ phenotype (Figure 4B).

**Figure 5:**
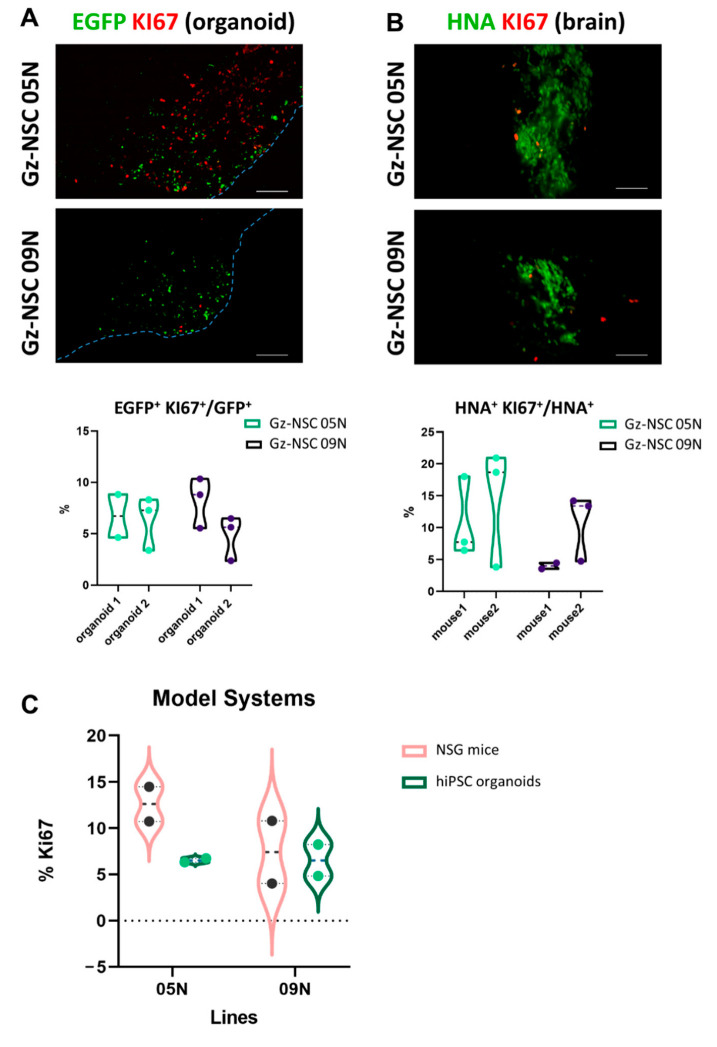
**Gz-NSC proliferation rate after transplantation in the human brain organoids and in mice brain.** (**A**) Gz-NSC proliferate within the brain organoids as shown by the expression of the proliferation marker KI67 in EGFP^+^ grafted cells 3 weeks after transplantation. Scale bar: 100 µm. (**B**) Gz-NSC proliferate at 3 weeks after transplantation in mice brain as shown by expression of the proliferation marker KI67 in HNA^+^ grafted cells. (**C**) Comparison of two Gz-NSC lines proliferation rate in human organoid versus mouse model.

Since proliferation capability is an important safety issue for the development of cell therapeutics, we studied the proliferation rate of the Gz-NSC lines when transplanted in the human and mice models. In organoids, quantification of Ki67^+^ transplanted cells showed a remarkably similar proliferation activity for both lines (6.54% of 2179 NSC-EGFP^+^ cells and 6.54% of 1493 NSC-EGFP^+^ cells, respectively) (Figure 5A). Quantification of Ki67 expression in cells transplanted in mice revealed a variable proliferation rate (12.6% and 7.4%), with a similar inter-line (between groups) and inter-subject (within groups) variability (Figure 5B). The proliferation rate of transplanted cells was slightly higher in mice than in organoids for both Gz-NSC lines, although the differences were not statistically significant (Figure 5C).

## 4. Discussion

In this work, we have explored whether human brain organoids can be used as in vitro models for cell transplantation. We have successfully transplanted NSC lines into brain organoids by injection and compared these data with standard xenograft studies in the brain of NSG mice. NSC proliferation and differentiation potential were similar in both models, suggesting that brain organoids can be informative when studying the proliferative and differentiation potential of cell products, which are important safety and efficacy indicators. Our results suggest that brain organoids can help reduce the number of animals used for regulatory efficacy and safety studies in cell therapy and, importantly, to facilitate preclinical studies.

The proliferation activity of transplanted cells was more uniform in brain organoids than in mouse brains, probably reflecting the higher complexity of the animal as a model system, with many factors affecting the reproducibility of the outcome. In this regard, brain organoids may be advantageous for addressing some specific aspects of NSC biology.

Our brain organoid model for cell transplantation would facilitate regulatory studies, since it is a relatively simple method that can be carried out in a cell culture cabinet without the need for highly regulated and specialized animal facilities. Other in vitro models for cell transplantation, such as brain slices, have been proposed, but that 3D model is usually generated from mice brains. Brain organoids have the advantage of being a human model, and provide more accurate information of human-specific cell–cell and cell–matrix interactions. Furthermore, unlike brain slices, organoids can be kept in culture for a longer time [25].

A limitation of our system is that, in the brain organoids, we cannot assess the host immune reaction to the cell graft, as the organoids lack a vascular and hematopoietic system, although with this protocol we can occasionally observe the presence of microglial cells in some organoids [29]. In animals, we can better evaluate the immunogenicity of our product, but we do not know if it will predict the reaction in immunocompetent hosts, as we usually employ immunodeficient or immunosuppressed animals. In this regard, the use of human brain organoids that consistently include microglial cells generated more recently [30] could be informative in evaluating the immune reaction to cell transplantation, and the interaction of the transplanted cells with the immune cells in the organoid.

Another limitation of our study is that our experiments have been performed with brain organoids generated by a default protocol; therefore, different brain structures and non-neural structures are formed. Furthermore, the generation of brain organoids is a variable process, with significant batch-to-batch and organoid-to-organoid differences [31]. Attempts are being made in the scientific community to decrease this variability and to automate the process. The use of small molecules to generate organoids of a specific brain structure (e.g., cerebral cortex) with no contamination with non-neural tissues can normalize identity and decrease variability [32], probably being a more robust tool to systematically evaluate differentiation and maturation of the NSC progeny. Finally, a technical consideration is that, for the transplantation into organoids, we used EGFP-transduced cells, and it is known that viral transduction can affect cell proliferation [33]. This can lead to an underestimation of the proliferative state of transplanted cells. Nevertheless, our preliminary findings might help guide future studies on the evaluation of safety and efficacy of cell therapeutics using human in vitro models.

Overall, our results suggest that the use of brain organoids as in vitro human models for study the safety and efficacy profile of new cell therapeutics is feasible and may help reduce animal experimentation. This provides unprecedented opportunities for elucidating mechanisms and studying cell integration in neural circuits. However, future studies should determine key factors for performing an effective transplantation, such as the age of the organoid in which the transplant is most effective and the optimal volume and number of transplanted cells. Furthermore, larger experiments will be required to validate the system and discern whether brain organoids increase predictability for future treatments.

## 5. Conclusions

NSC differentiate into neuroblasts and oligodendrocyte precursors and generate few astrocytes in both human brain organoids and mouse brains. Similar proliferation potential was detected in both models, although it was more variable in animals. Overall, our results suggest that brain organoids can be useful in the evaluation of cell therapy approaches, facilitating preclinical studies and helping to reduce the number of animals used for testing, being aligned with the philosophy of the 3Rs.

## Data Availability

The data that support the findings of this study are available upon reasonable request to the corresponding author.

## Supplementary Materials

The following supporting information can be downloaded at: https://www.mdpi.com/article/10.3390/ani12223150/s1, Figure S1: iPSC and organoid quality controls; Table S1: List of antibodies used.
