# Supplementary material for: Brain Organoids to Evaluate Cellular Therapies
Source: Animals (Basel). 2022 Nov 15;12(22):3150. doi: 10.3390/ani12223150 (PMC9686900; doi:10.3390/ani12223150)
Supplement: Supplementary file 1 [file animals-12-03150-s001.zip › animals-1984526-supplementary.pdf]

Fig. S1 (to Fig.1). TOP

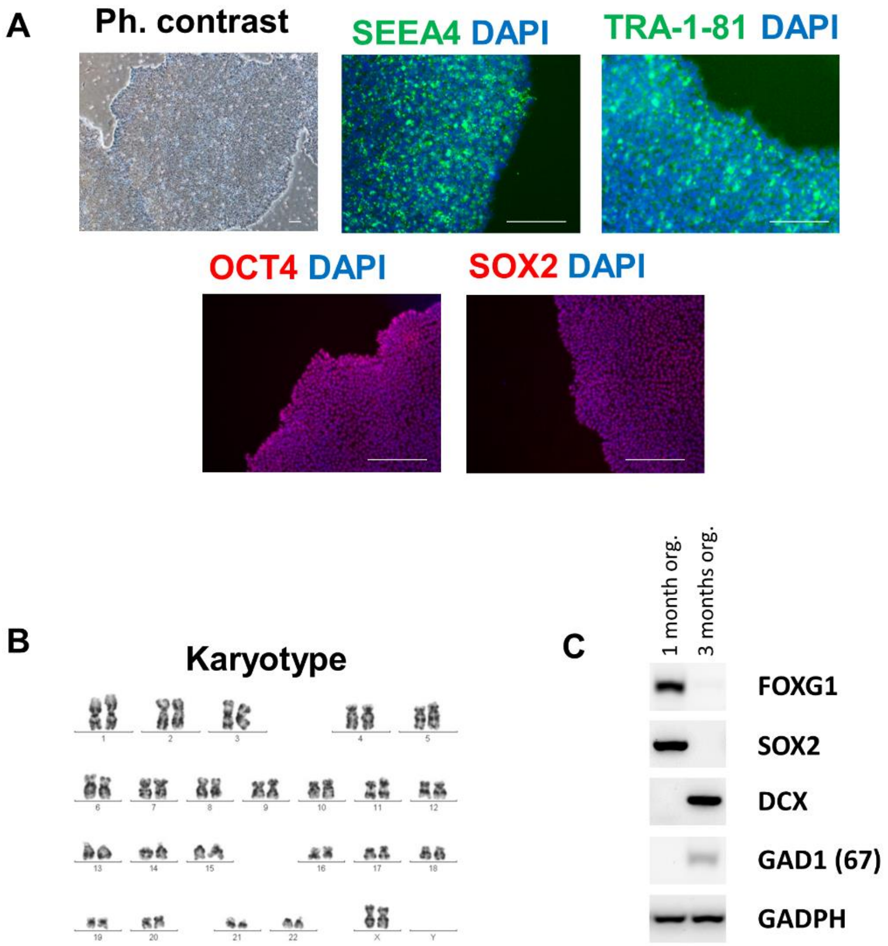

Figure S1: iPSC and organoid quality controls. A) Phase contrast image of iPSC colonies and expression of the pluripotency markers SEEA4, TRA-1-81, OCT4 and SOX2 prior organoid generation. Scale bar: 100 µm. B) iPSC used for organoid generation showed normal karyotype. C) Analysis of different markers expressed during human brain development at different time points of organoid culture.

Table S1. List of antibodies used.

Primary antibodies	Dilution	Company (Cat)
Olig2	1:500	R&D System, Minneapolis, Minnesota, USA (AF2418)
Tuj1 (β-III Tubulin)	1:1000	Covance, Princeton, New Jersey, USA (MMS-43SP)
MAP2	1:100	Santa Cruz Biotechnology, Dallas, USA (sc-32791)
SATB2	1:100	Abcam, Cambrigde, UK (ab34735)
CTIP2	1:100	Abcam, Cambrigde, UK (ab18465)
TUBβIV (β-IV Tubulin)	1:100	Sigma, San Luis, Misuri, USA (T7941)
FOXJ1	1:500	Abcam, Cambrigde, UK (ab18259)
TTR (Transthyretin)	1:200	Agilent Technologies, Santa Clara, CA, USA (A0002)
GFP Rabbit	1:200	Invitrogen, Waltham, Massachusetts, USA (A11122)
GFP Chicken	1:200	Abcam, Cambrigde, UK (ab13970)
Ki67	1:100	Dako by Agilent Tech, Santa Clara, CA, USA (M7240)
Nestin	1:1000	Abcam, Cambrigde, UK (ab22035)
DCX (Doublecortin)	1:1000	Millipore, Burlington, Massachusetts, USA (AB2253)
GFAP Rabbit	1:500	Millipore, Burlington, Massachusetts, USA (AB5804)
GFAP Chicken	1:1000	Abcam, Cambrigde, UK (ab4674)
HNA (Human Nuclei)	1:200	Abcam, Cambrigde, UK (ab191181)
IBA1	1:200	Abcam, Cambrigde, UK (ab5076)
Ku80	1:100	Cell Signaling Tech, Danvers, Massachusetts, USA (#2753)
hGFAP (human GFAP)	1:500	Takara Bio, Shiga, Japan (Y40420)
SSEA4	1:100	Cell Signaling Tech, Danvers, Massachusetts, USA (MC813)
Tra-1-81	1:100	Millipore, Burlington, Massachusetts, USA (MAB4381)
OCT4	1:400	Cell Signaling Tech, Danvers, Massachusetts, USA (C30A3)
SOX2	1:500	Millipore, Burlington, Massachusetts, USA (AB5603)
NKX2.1	1:100	R&D System, Minneapolis, Minnesota, USA (MAB94581)
**Secondary antibodies**	**Dilution**	**Company (Cat)**
Donkey anti-Rabbit IgG (H+L) (Alexa Fluor 488, Green)	1:500	Invitrogen, Waltham, Massachusetts, USA (A21206)
Donkey anti-Mouse IgG (H+L) (Alexa Fluor 488, Green)	1:500	Invitrogen, Waltham, Massachusetts, USA (A21202)
Donkey anti-Guinea pig IgG (H+L) (Alexa Fluor 488, Green)	1:400	Jackson InmunoR, Cambrigde, UK (706-454-148)
Donkey anti-Rat IgG (H+L) (Alexa Fluor 488, Green)	1:500	Invitrogen, Waltham, Massachusetts, USA (A48269)
Donkey anti-Rabbit IgG (H+L) (Alexa Fluor 594, Red)	1:500	Invitrogen, Waltham, Massachusetts, USA (A21207)
Donkey anti-Goat IgG (H+L) (Alexa Fluor 594, Red)	1:500	Invitrogen , Waltham, Massachusetts, USA (A11058)
Donkey anti-Chicken IgG (H+L) (Alexa Fluor 594, Red)	1:400	Jackson InmunoR, Cambrigde, UK (703-585-155)
